# Absence of Resistance Mutations in the Integrase Coding Region among ART-Experienced Patients in the Republic of the Congo

**DOI:** 10.3390/microorganisms9112355

**Published:** 2021-11-15

**Authors:** Ferdinand Emaniel Brel Got, Patricia Recordon-Pinson, Ghislain Loubano-Voumbi, Dagene Ebourombi, Marie-Lise Blondot, Mathieu Metifiot, Gontran Ondzotto, Marie-Line Andreola

**Affiliations:** 1 Faculté des Sciences de la Santé, Université Marien Ngouabi, Brazzaville BP69, Democratic Republic of the Congo; emmanueldeferdinand@gmail.com (F.E.B.G.); ghisloubano@yahoo.fr (G.L.-V.); debourombi@gmail.com (D.E.); gontranondzotto@gmail.com (G.O.); 2 UMR 5234 Microbiologie Fondamentale et Pathogénicité, CNRS, Univ. Bordeaux, F-33000 Bordeaux, France; marie-lise.blondot@u-bordeaux.fr (M.-L.B.); mathieu.metifiot@u-bordeaux.fr (M.M.); marie-aline.andreola@u-bordeaux.fr (M.-L.A.); 3 Virology Laboratory, WHO HIV Center, CHU Bordeaux, F-33000 Bordeaux, France

**Keywords:** HIV, resistance, the Republic of the Congo

## Abstract

Background: HIV infects around one hundred thousand patients in the Republic of the Congo. Approximately 25% of them receive an antiretroviral treatment; current first-line regimens include two NRTIs and one NNRTI, reverse transcriptase inhibitors. Recently, protease inhibitors (PIs) were also introduced as second-line therapy upon clinical signs of treatment failure. Due to the limited number of molecular characterizations and amount of drug resistance data available in the Republic of the Congo, this study aims to evaluate the prevalence of circulating resistance mutations within the *pol* region. Methods: HIV-positive, ART-experienced patients have been enrolled in four semi-urban localities in the Republic of the Congo. Plasma samples were collected, and viral RNA was extracted. The viral load for each patient was evaluated by RT-qPCR, following the general diagnostic procedures of the University Hospital of Bordeaux. Finally, drug resistance genotyping and phylogenetic analysis were conducted following Sanger sequencing of the *pol* region. Results: A high diversity of HIV-1 strains was observed with many recombinant forms. Drug resistance mutations in RT and PR genes were determined and correlated to HAART. Because integrase inhibitors are rarely included in treatments in the Republic of the Congo, the prevalence of integrase drug resistance mutations before treatment was also determined. Interestingly, very few mutations were observed. Conclusions: We confirmed a high diversity of HIV-1 in the Republic of the Congo. Most patients presented an accumulation of mutations conferring resistance against NRTIs, NNRTIs and PIs. Nonetheless, the absence of integrase mutations associated with drug resistance suggests that the introduction of integrase inhibitors into therapy will be highly beneficial to patients in the Republic of the Congo.

## 1. Introduction

**Figure 1:**
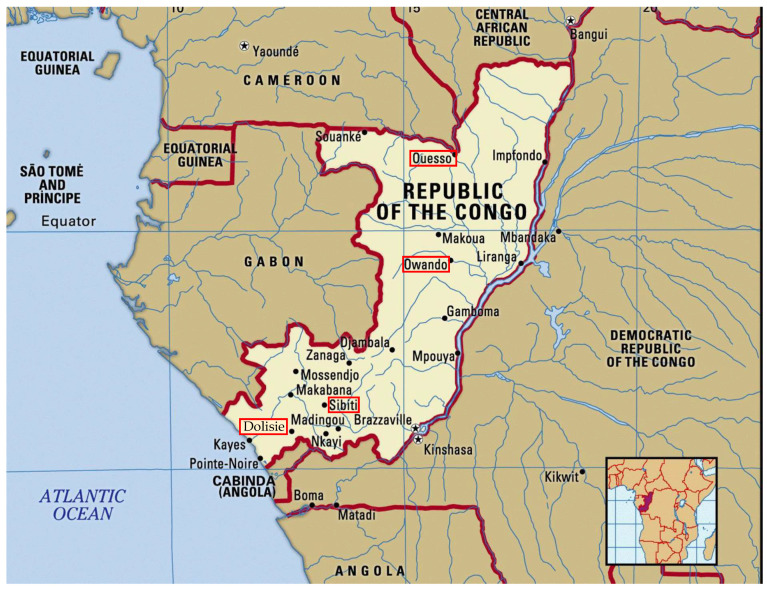
Map of the Republic of the Congo indicating the four localities of the study: Ouesso, Owando, Dolisie and Sibiti.

Of the 38 million people worldwide currently living with HIV, Sub-Saharan Africa (SSA) remains the most affected region, accounting for approximatively 70% of all people living with HIV (UNAIDS 2020). In 2020, UNAIDS estimated that one hundred thousand people were living with HIV in the Republic of the Congo (RC), and only 25% of them received an antiretroviral treatment (ART). According to the 2016 WHO guidelines, a combination of two nucleoside reverse transcriptase inhibitors (NRTIs) and one non-nucleoside reverse transcriptase inhibitor (NNRTI) is recommended as a first-line regimen, and two NRTIs and one protease inhibitor (PI) as second-line therapy. Recently, Dolutegravir (DTG), an integrase strand transfer inhibitor (INSTI), was introduced as first-line therapy, according to the WHO guidelines. ART efficacy requires regular biological and clinical monitoring of ART-experienced patients in order to monitor the appearance of resistance. Despite the efficacy of these combination therapies, some patients still experience treatment failure. The monitoring of HIV-1-infected Congolese living in rural localities remains problematic and could affect ART efficacy. In these localities, HIV-infected populations face several challenges that impact adherence to treatment and facilitate emerging resistance mutations, such as distance to medical structures, lack of infrastructure for biological monitoring (an absence of routine CD4 counts, viral load measurements or genotyping), treatment breaks, stigma and cultural beliefs. There are limited data available on the molecular characterization of HIV-1 in the RC. Studies performed in urban regions before the inclusion of DTG reported the predominance of specific circulating recombinant forms (CRFs), including CRF02_AG, and an important genetic variability with the accumulation of drug resistance mutations (DRM) [1,2]. This study aims to evaluate the drug resistance profile of HIV-1 within the *pol* region, and describe the circulating strains among ART-experienced patients in four semi-rural localities in the RC (Figure 1).

## 2. Materials and Methods

### 2.1. Study Design, Populations and Context

From 2019 to 2020, we conducted a cross-sectional study of HIV-1-infected patients, excluding newly diagnosed patients, from four care centres in four semi-rural localities of the RC (regional hospitals in Ouesso and Owando in the north, and regional hospitals in Dolisie and Sibiti in the south of the country, Figure 1). All these centres provide medical consultations and ARV supplies free of charge to patients, except for biological monitoring. Therefore, due to the lack of virological and immunological monitoring in these regions (no viral load or CD4 count), the selection concerned only patients with clinical failure according to the WHO recommendations. Clinical and sociodemographic data were obtained from record centres. All patients were previously diagnosed with HIV-1 using the national testing algorithm, which included two rapid assays, and were all ART-experienced.

### 2.2. Ethical Considerations

This study was approved by the ethics committee for research in health sciences of the Ministry of Scientific Research and Technology. Permission to access public sector health facilities was obtained from each regional Department of Health. Before inclusion, fully informed consent was obtained from each patient, and informed consent was obtained from the parents or legal guardians of younger patients.

### 2.3. Biosample Collection and Molecular Analysis

For each patient included, approximately 4 mL of venous blood was collected into an ethylenediaminetetraacetic acid (EDTA) tube. The plasma samples (2 mL per patient) obtained after centrifugation were aliquoted in cryotubes, then stored at −80 °C before being transported by flight for molecular analysis to the UMR5234 Fundamental Microbiology and Pathogenicity Laboratory of Bordeaux University. HIV-1 plasma viral load was evaluated by RT-qPCR with the COBAS^®^ 6800 System, version 1.04, according to the manufacturer’s instructions, with a limit of detection of 20 copies/mL at the biomolecular platform of the University Hospital of Bordeaux (CHU). Viral RNA was extracted from plasma samples using the High Pure Viral RNA Kit (Roche Diagnostics Systems^®^, Nutley, State of New Jersey, USA), following the manufacturer’s protocol, and stored at −80 °C until further use. For the identification of HIV-1 subtype and drug-associated resistance mutations, reverse transcriptase (RT), protease (PR), and integrase (INT) coding regions were amplified according to the protocol of the French National Agency for AIDS Research (ANRS, available online: http://www.hivfrenchresistance.org, accessed on 1 March 2021 (Version January 2015)). PCR products were processed by Sanger sequencing on both strands using an Applied Biosystems 3500xl Dx Genetic Analyzer. Accordingly, minority variants could not be detected and only mutations present at minimum frequencies above 15% to 20% of the viral quasi-species were monitored. FASTA sequences of each region (PR, RT and INT) were assembled using the Integrative Database Network System (IDNS, version v3_11_0r4(r32389) Smartgene 2021) in order to determine the HIV-1 genotype and drug resistance mutations. PR, RT and INT sequences were analyzed for DRMs according to the ANRS algorithm (available online: http://www.hivfrenchresistance.org/ (December 2020 - Version n°31, accessed on 1 March 2021)) and the WHO international working group for the surveillance of transmitted HIV-1 drug resistance [3]. The derived nucleotide sequences were aligned by the ClustalW2 version 2.1 alignment program with known reference strains of M, N, and O pooled from the HIV-1 gene databank (available online: http://hiv.lanl.gov/ (2019), accessed on 1 March 2021). Phylogenetic trees were inferred using the neighbor-joining method from matrix distances calculated after gap stripping alignments, according to a Kimura two-parameter algorithm. Bootstrapping was performed with 100 replicates. The circular trees were obtained using ITOL (available online: http://itol.embl.de/ [4], accessed on 1 March 2021). Accession numbers to the GenBank database for sequenced HIV-1 strains were OK180621-OK180687, OK180688-OK180764 and OK180765-OK180811 for protease, reverse transcriptase and integrase genes, respectively. The genotypic susceptibility score (GSS), which estimates the number of HIV-active drugs in any given combination ART regimen, was calculated for each molecule of the treatment as follows: (1) susceptible and potential low-level resistance was scored as 1, (2) low-level and intermediate resistance were pooled as intermediate and scored as 0.5, and (3) high-level resistance was scored as 0. The GSS was assigned by summing the individual resistance scores for each line drug in the combination ART regimen [5].

### 2.4. Statistical Analysis

Analyses were performed with R version 3.4.4 and GraphPad Prism 6 (GraphPad, San Diego, CA, USA) software. Quantitative variables were expressed as the median and interquartile range (IQR) and compared using the one-way ANOVA test. Categorical variables were presented as number of cases and percentages, and were compared using Pearson’s chi-squared test. A *p* value of < 0.05 was considered statistically significant.

## 3. Results

### 3.1. Characteristics of the Study Population

We included 210 patients in this study. Only samples from 85 patients were successfully amplified, consisting of 29 samples from Dolisie, 33 from Ouesso, 13 from Owando, and 10 from Sibiti (Figure 1). Among this cohort, the median age was 44 ± 14 years and 63.5% were female (Table 1). More than 75% of patients had a high school education or higher education, and about 40% were in a relationship. According to the WHO HIV clinical staging, patients were overall equally distributed between stages 2, 3 and 4. A significant difference (*p* = 0.02) was noticed in the localities of Dolisie and Owando, where more patients in stage 4 were classified. In addition, 35.3% of patients had a viral load above 100,000 copies/mL. The majority of patients (47.1%) were on first-line regimen AZT + 3TC + NVP or EFV, and only 17.6% of the patients received a DTG-containing regimen, an integrase strand transfer inhibitor (INSTI). Finally, 42.4% of patients were on ART for more than 48 months.

### 3.2. HIV Genotypic Subtypes

**Figure 2:**
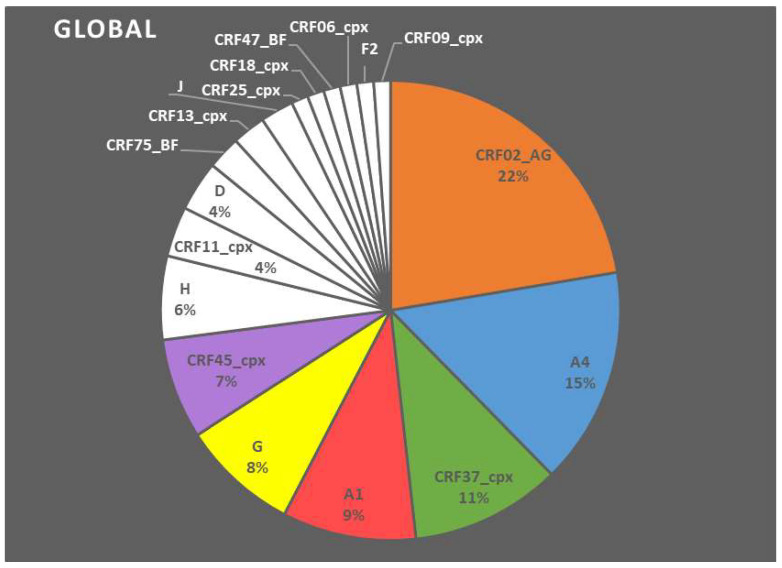
Global analysis of the HIV subtypes diversity according to the genotyping of the *pol* region. CRF means circulating recombinant form.

From the 85 samples that were successfully amplified, subsequent Sanger sequencing was performed. Altogether, 76 samples were sequenced for the RT region, 68 for the PR, and 46 for the INT. The global analysis of this *pol* region showed that HIV-1 CRF02_AG was the predominant subtype with a prevalence of about 22% (Figure 2), followed by subtype A4 (15%). We also observed high diversity, including pure subtypes and recombinant forms in CRF37_cpx (11%), A1 (9%), G (8%), CRF45_cpx (7%), H (6%), CRF11_cpx (4%), D (4%), and in subtypes with less than 4%, CRF75_BF, CRF13_cpx, J, CRF25_cpx, CRF18_cpx, CRF47_BF, CRF06_cpx, F2 and CRF09_cpx. The analysis by localities confirmed the predominance of CRF02_AG in all sub-localities. Slight differences in the distribution of the recombinant forms are observed. For example, CRF13_cpx represented 15% of the circulating strains in Owando, whereas A4 was predominant (19%) in Ouesso. However, due to the limited number of samples, it was not possible to determine whether a locality had a statistically different subtype representation compared to global analysis.

### 3.3. Drug Resistance Mutations (DRMs)

As expected, and according to the treatment of the patients and the viral load measured, we observed the presence of drug resistance mutations for all patients.

#### 3.3.1. NRTI and NNRTI

**Figure 3:**
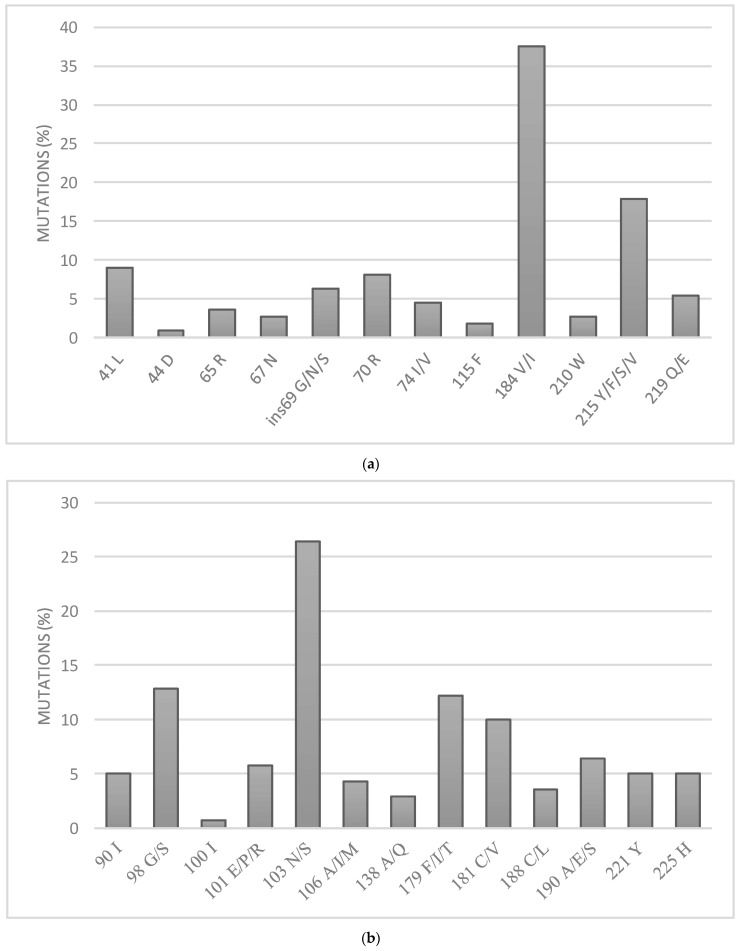
Observed frequency of mutations conferring resistance to (**a**) nucleoside reverse transcriptase inhibitors (NRTIs) and (**b**) non-nucleotide reverse transcriptase inhibitors (NNRTIs).

The most frequent mutations conferring resistance to nucleoside reverse transcriptase inhibitors (NRTIs) were M184V/I with a prevalence of 37%, followed by T215Y/F/S/V at 18% (Figure 3a). Additional mutations were also observed at lower frequencies such as M41L (9%), K70R (8%), K219Q/E (5%), L74I/V (4%), K65R (4%), L210W (3%), D67N (3%), and Y115F (2%). We also observed insertions in position 69 (ins69 G/N/S).

Regarding resistance to non-nucleoside reverse transcriptase inhibitors (NNRTIs), observed frequent mutations included K103N (26%), A98G/S (13%), V179F/I/T (12%), and Y181C/V (10%). The additional mutations at lower frequencies were G190A/E/S (6%), K101E/P/R (6%), P225H (5%), V90I (5%), H221Y (5%), V106A/I/M (4%), Y188C/L (4%), E138A/Q (3%), and L100I (1%). (Figure 3b).

#### 3.3.2. PI and INSTI

**Figure 4:**
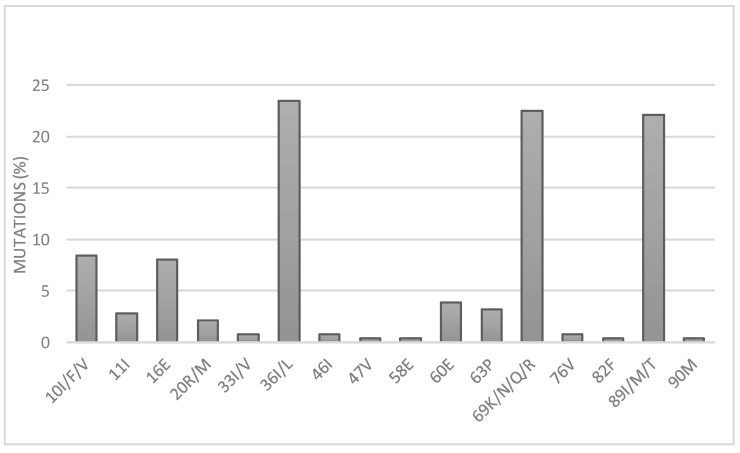
Observed frequency of mutations conferring resistance to protease inhibitors (PIs).

Because protease inhibitors were not often used (4.7%, Table 1), the major PI mutations responsible for drug resistance (I47V and L76V) were mostly rare and accounted for at less than 5%. However, accessory mutations were more frequent, and were distributed as follows: M36I/L (24%), H69K/N/Q/R (22%), L89I/M/T (22%), L10I/F/V (8%), G16E (8%), and with less than 5%, V11I, K20R/M, L33I/V, M46I, Q58E, D60E, L63P V82F and L90M (Figure 4).

**Figure 5:**
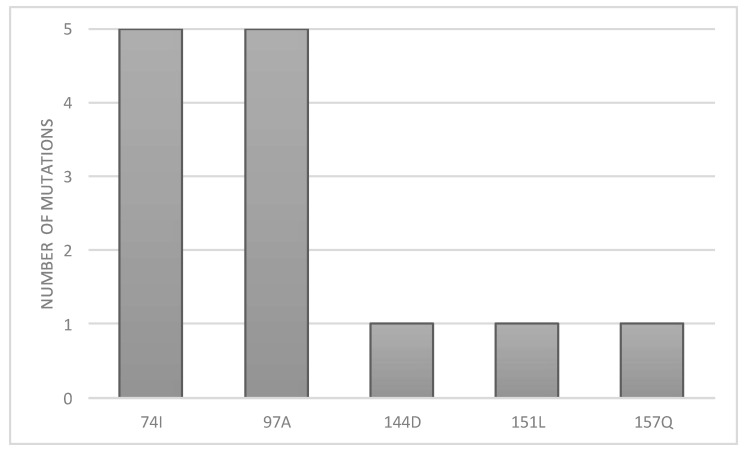
Observed frequency of mutations conferring resistance to integrase strand transfer inhibitors (INSTIs).

Interestingly, even if 17.6% of the patients received DTG as part of their regimen, no major INSTI mutation was observed. Still, T97A (38%) and L74I (38%) were abundantly selected (Figure 5). Even though these two mutations are naturally polymorphic, they are associated with DTG resistance when associated with other mutations, such as Q148. Additionally, mutations N144D, V151L and E157Q were less frequently observed, with a prevalence of 5% each.

#### 3.3.3. GSS

**Figure 6:**
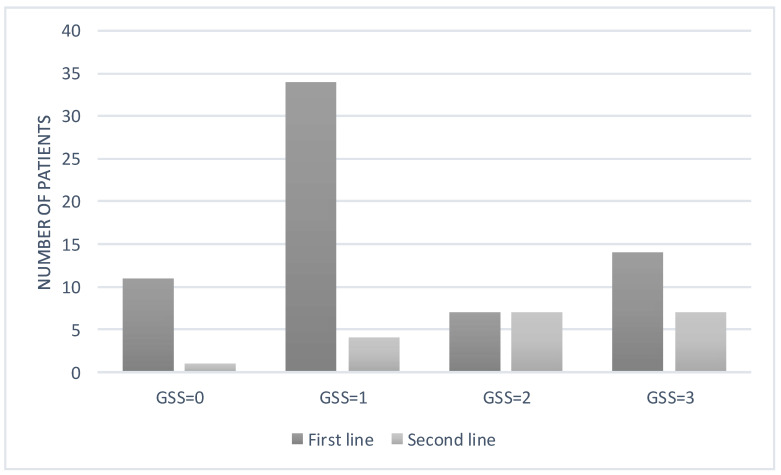
Genotypic susceptibility scores (GSSs) to the current first-line and second-line ART therapy of the patients.

For the calculation of the GSS, we classified different types of treatment into two lines. The first-line regimen included combinations based on two NRTIs and one NNRTI, and the second-line regimen included combinations based on PI or DTG according to national therapeutic guidelines. Based on the GSS estimates, 34 patients had a GSS = 1, meaning that they harboured genotypes that were not fully susceptible to all three drug components of the preferred first-line regimen (Figure 6). Interestingly, if we considered the second-line regimen based on PI or DTG, the GSS score was mostly 2 or 3.

## 4. Discussion

Limited access to HIV drug resistance testing in the RC is a major barrier to monitoring the effectiveness of combination therapies, especially at a time when DTG is being introduced into treatment lines. It is therefore very important to know the resistance mutations circulating in the RC in order to better orientate therapeutic choices. This is the first study from the Republic of the Congo to characterize HIV-1 genetic subtypes and HIV drug resistance in ART-experienced patients from four semi-rural localities.

This study aimed to evaluate the prevalence of resistance mutations within the *pol* region. It confirms the genetic diversity of HIV-1 in the RC, and shows that genetically diverse HIV-1 strains are observed in the RC. We noticed a decrease in the proportion of pure subtypes with an increase in the number of CRFs (Figure 2), whereas previous work showed a high diversity of HIV-1 with a majority of undetermined recombinant forms (URFs) followed by CRF02_AG, CRF37_cpx, G, A1, B, D, H [6,7,8]. Our results show that CRF02_AG (22%) was the most prevalent subtype, in agreement with previous data [1]. Indeed, CRF02_AG is one of the most prevalent CRFs in the global HIV-1 epidemic, and is the major circulating recombinant form in West and Central Africa today [9,10]. According to previous studies, CRF02_AG was observed as the second most predominant genotype in the Republic of the Congo (URF, undetermined recombinant forms, was the most predominant) and the most prevalent genotype in Gabon, a neighboring country [2,11]. The distribution of genotypes in our study was almost identical to that found in Cameroon [12]. This can be explained by the fact that both countries share land borders, with the possibility of movement between regions, especially for sellers.

As expected, high prevalence of DRM to NRTIs and NNRTIs were found as a consequence of virological failure, and can explain why 35.3% of patients have a viral load of up to 100,000 copies/mL. The detection of these DRMs to NRTIs and NNR largely concerned patients on first-line regimen (composed essentially of these drugs). Once more, this indicates that prolonged drug exposure without viral load monitoring, which is necessary for early detection of virological failure, is a major drawback in the fight against drug resistance. Unfortunately, biological monitoring is lacking in these semi-rural settings, and HIV patients are inadvertently maintained on their “failing” drug regimens with the consequence of the accumulation of drug resistance mutations. To avert the situation, assays need to be developed for biological patient monitoring, including CD4 counts, viral load and drug resistance testing, which are key parameters for global HIV care in semi-rural areas.

The M184V mutation was the most frequent NRTI DRM. This coincided with the extensive use of 3TC (or FTC) as a backbone in all regimens in the RC and all over SSA. The presence of this mutation significantly reduces 3TC activity, while conferring low-level resistance to ABC, and reduces viral replicative fitness [13,14,15]. In contrast, the M184V mutation increases susceptibility to TDF and AZT, while simultaneously limiting the emergence of drug resistance to both molecules [16,17]. Furthermore, 3TC has a low genetic barrier. The major NNRTI resistance mutation identified was the K103N, which is known to decrease the binding affinity of EFV and NVP to the viral target, resulting in resistance to antiretroviral drugs and increased risk of virological failure [18,19]. Unlike M184V, K103N has no negative impact on viral fitness.

The observed high levels of DRMs to NRTI and NNRTIs in these semi-rural localities could be linked to several factors. Firstly, the availability of transportation to treatment centers is generally significantly reduced in rural areas compared to urban areas. Secondly, shortages of ART supplies are common occurrences in rural localities. Finally, cultural differences and stigma hinder patients from easily undertaking their treatment, alongside inadequate planning and poor follow-up (clinical, biological and psychological) from health authorities. The majority of the NRTI and NNRTI resistance mutations found in this study are quite similar to ones previously described by Pircher et al. [1], who reported data on HIV resistance to drugs in Brazzaville. This suggests that viral strains bearing similar resistance mutations are circulating between different localities in the RC.

Despite the high prevalence of DRMs to NRTIs and NNRTIs, the prevalence of DRMs to PI and INSTIs was relatively low or absent. However, many polymorphic mutations were observed. These results confirm the situation described in the WHO 2019 HIV drug resistance report [20]. In the RC, DTG, an INSTI, was recommended in 2020 but is currently not available all over the country. In this study, we found polymorphic accessory INSTI mutations, but we did not observe any primary INSTI drug-resistant mutations. These findings support the notion that DTG may be safe to use in INSTI-naïve settings [21,22]. Despite its potency, good tolerability and high genetic barrier to developing drug resistance, growing use of DTG in SSA warrants the need to closely monitor the appearance of primary resistance mutations to DTG and the occurrence of treatment failures [23]. Indeed, in a region with high HIV-1 subtype diversity, it is important to understand the impact of circulating HIV-1 subtypes on the efficacy of DTG. It is important to point out that our analyses were performed using routine Sanger sequencing that does not allow for the detection of minority viral variants that comprise less than 20% of the viral population in a patient sample [24].

Another limitation arose from the lack of data on treatment adherence. Accordingly, it was not possible to associate the observed virological failure to poor adherence. Importantly, the lack of CD4 counts did not allow us to assess the immunological response of patients to different ART regimens. In addition, the sample size was small in some localities, and the rate of amplification and sequencing of the three target genes further reduced the numbers used in the statistical analysis. Even though 210 patients were included in this study, only 85 samples were successfully processed. The thawing and refreezing cycles of plasma during travel from the localities to Bordeaux could have favored the degradation of nucleic acids in certain samples, which could explain the low amplification rate of the samples. Caution should be taken not to overinterpret those data, and further characterization should be performed to evaluate the global HIV-infected population of the RC. Nonetheless, even if this study presented no evidence that a duration of drug exposure without viral load monitoring will lead to an accumulation of DRMs, our findings agree with the World Health Organization, which recommends routine viral load monitoring for the detection of treatment failure in patients on ART. Therefore, the true level of DRM might be underestimated, so epidemiological studies should be conducted in the general HIV-1-infected population each year in order to monitor the circulating subtypes of the HIV-1 strains, and to evaluate the emergence of new ARV DRMs.

## 5. Conclusions

Our findings highlight the potential consequences for molecular follow-up of HIV-1-infected patients. Without proper biological monitoring, we observed that viruses replicated actively in many patients, with a selection of drug resistance mutations among viral strains present in the RC. It also highlights the tremendous benefits of the introduction of DTG in therapeutic lines, according to the absence of major INSTI DRMs. As mutation and recombination are the major forces driving HIV-1 genomic diversity, the continual emergence of new recombinant forms presents an enormous surveillance challenge. Thus, implementing a national HIV-1 DRM surveillance program is critical to help guide ART choice and improve interventional strategies.

## Figures and Tables

**Table 1:** Baseline characteristics (sociodemographic, clinical and biological) of the study population in general, and in their respective localities.

Characteristics	Global N = 85 (%)	Dolisie N = 29 (%)	Ouesso N = 33 (%)	Owando N = 13 (%)	Sibiti N = 10 (%)	*p*-Value
**Gender**						0.36
Male	31 (36.5)	14 (48.3)	9 (27.3)	5 (38.5)	3 (30.0)	
Female	54 (63.5)	15 (51.7)	24 (72.7)	8 (61.5)	7 (70.0)	
**Age, years**	44 (6–72)	47 (18–72)	42 (6–62)	42 (14–72)	46 (23–72)	0.61
Mean (min–max)						
**Educational level**						0.14
grade school	19 (22.4)	5 (17.2)	10 (30.3)	3 (23.1)	1 (10.0)	
high school	58 (68.2)	18 (62.1)	21 (36.6)	10 (76.9)	9 (90.0)	
university level	8 (9.4)	6 (20.7)	2 (6.1)	-	-	
**Marital status** in a relationship	37 (43.5)	11 (37.9)	15 (45.5)	6 (46.2)	5 (50.0)	
single	24 (28.2)	9 (31.0)	7 (21.2)	3 (23.1)	5 (50.0)	
divorced	18 (21.2)	7 (24.1)	8 (24.2)	3 (23.1)	-	
widower	6 (7.1)	2 (6.9)	3 (9.1)	1 (7.7)	-	
**WHO HIV clinical staging**						**0.02**
stage 1	6 (7.1)	1 (3.4)	5 (15.2)	-	-	
stage 2	28 (32.9)	7 (24.1)	12 (36.4)	4 (30.8)	5 (50.0)	
stage 3	26 (30.6)	8 (27.6)	13 (39.4)	2 (15.4)	3 (30.0)	
stage 4	25 (29.4)	13 (44.8)	3 (9.1)	7 (53.8)	2 (20.0)	
**Current ART regimens**						0.07
AZT+3TC + (NVP or EFV)	40 (47.1)	12 (41.4)	17 (51.5)	4 (30.8)	7 (70.0)	
TDF + 3TC + EFV	26 (30.6)	5 (17.2)	11 (33.3)	7 (53.8)	3 (30.0)	
DTG+3TC + (ABC or TDF)	15 (17.6)	10 (34.5)	3 (9.1)	2 (15.4)	-	
(ABC or AZT) + 3TC + LPV/r	4 (4.7)	2 (6.9)	2 (6.1)	-	-	
**Duration of ART exposure (months)**						0.91
Median						
≤24	9 (10.6)	2 (6.9)	4 (12.1)	2 (15.4)	1 (10.0)	
25–36	12 (14.1)	4 (13.8)	5 (15.2)	2 (15.4)	1 (10.0)	
36–48	28 (32.9)	10 (34.5)	8 (24.2)	6 (46.2)	4 (40.0)	
≥48	36 (42.4)	13 (44.8)	16 (48.5)	3 (23.1)	4 (40.0)	
**Viral load cells/mm^3^**						0.75
<1000	13 (15.3)	3 (10.3)	7 (21.2)	3 (23.1)	-	
1000–10,000	15 (17.6)	5 (17.2)	6 (18.2)	1 (7.7)	3 (30.0)	
10,000–100,000	23 (27.1)	9 (31.0)	6 (18.2)	4 (30.8)	4 (40.0)	
>100,000	30 (35.3)	10 (34.5)	12 (36.4)	5 (38.5)	3 (30.0)	
ND	4 (4.7)	2 (6.9)	2 (6.1)	-	-	

Abbreviations: ART: antiretroviral therapy; AZT: zidovudine; 3TC: lamivudine; NVP: nevirapine; EFV: efavirenz; TDF: tenofovir; DTG: dolutegravir; ABC: abacavir; LPV/r: lopinavir/ritonavir; ND: not determined.

## Data Availability

Accession numbers to the GenBank database for sequenced HIV-1 strains were OK180621-OK180687, OK180688-OK180764 and OK180765-OK180811 for protease, re-verse transcriptase and integrase genes, respectively.
